# Biventricular Impella (Bi-Pella) in Refractory Cardiogenic Shock: The First Case from the Middle East

**DOI:** 10.1155/2021/6676339

**Published:** 2021-07-22

**Authors:** Abdulaziz Almejren, Abdullah Alenezi

**Affiliations:** Chest Disease Hospital, Ministry of Health, Kuwait, Kuwait

## Abstract

Cardiogenic shock (CS) associated with biventricular failure (BiVF) carries significant in-hospital morbidity and mortality. We describe here the successful use of percutaneous biventricular Impella (Bi-Pella) for cardiogenic shock secondary to acute biventricular myocardial infarctions (AMI-CS), as guided by parameters such as mixed venous oxygen saturation (SvO2), pulmonary artery pulsatility index (PAPi), central venous pressure (CVP), and cardiac power output (CPO). We aim to highlight the promising outcomes of timely implanted biventricular Impella in (AMI-CS).

## 1. Introduction

Cardiogenic shock is responsible for a significant percentage of morbidity and mortality after acute MI. However, early identification of cardiogenic shock and the early use of mechanical circulatory support (MCS) result in favorable outcomes. The Detroit Cardiogenic Shock Initiative described that patients with AMI and cardiogenic shock were supported with Impella (Abiomed, Inc., Danvers, Massachusetts, USA) before PCI resulted in survival to device removal of 89% and survival to discharge of 84% [1]. We present a case of AMI-CS in which a patient underwent insertion of an intra-aortic balloon pump (IABP) and associated coronary revascularization. Despite this, he experienced resistant cardiogenic shock that necessitated Impella CP and subsequent Impella RP, which resulted in a dramatic improvement in his hemodynamic parameters and overall condition.

## 2. History of Presentation

A 43-year-old man transferred from a non-PCI-capable hospital with acute onset chest pain for 4 h. He had no associated shortness of breath, cough, or fever. On examination, his blood pressure was 100/58 mm Hg, pulse rate was 120 beats/min and regular, respiratory rate was 20 breaths/min, oxygen saturation was 96% on room air, and his temperature was 37.2°C. He was fully conscious and had cold extremities. Cardiovascular examination revealed normal heart sounds and jugular venous distension. Lungs were clear with good air movement bilaterally, and the rest of the systemic examination was unremarkable.

### 2.1. Learning Objective

Case: a patient who presented with resistant cardiogenic shock secondary to acute myocardial infarction.
  1. We consider the early use of biventricular Impella in resistant cardiogenic shock secondary to acute myocardial infarction.

### 2.2. Medical History

The patient did not have any past medical history.

### 2.3. Differential Diagnosis

The differential diagnosis of acute chest pain without hypoxia in such a 43-year-old man is broad. A typical presentation of acute coronary syndrome was a major concern. Other differential diagnoses include acute heart failure and acute pulmonary embolism (PE).

### 2.4. Investigations

Results of the patient's laboratory tests were as follows: high-sensitivity (hs) cardiac troponin (cTn) was 2700 ng/l (normal range < 14 ng/L), mixed venous oxygen saturation (SvO2) was 40% (normal range 65–70%), and serum creatinine was 398 *μ*mol/L (baseline creatinine level was not available). An electrocardiogram showed ST-segment elevation in leads III, avF, and V3– V4 and new-onset right bundle branch block, and the rest of the systemic examination was unremarkable. A transthoracic echocardiogram revealed severe biventricular dysfunction, and the estimated left ventricular ejection fraction (LVEF) was 15%.

### 2.5. Management

He presented to a non-PCI capable hospital and was treated with fibrinolytic therapy. The patient was then transferred to our center for rescue PCI, which revealed chronic total occlusion (CTO) of mid-LAD, acute occlusion of the RCA, and critical occlusion of the left midcircumflex coronary artery. The operator inserted the intra-aortic balloon pump (IABP) and referred the patient to surgical revascularization.

A few hours later, the patient became more hypotensive, and we elected to proceed with PCI, with successful stenting to RCA, LCX, along with the insertion of Impella CP support. The mid-LAD (CTO) was left for a staged procedure. Despite successful coronary revascularization and Impella CP support, the patient developed overt cardiogenic shock, resistant to intravenous fluid and vasopressors. The next morning, repeat mixed venous oxygen saturation remained as low as 40%, and transthoracic echocardiography showed persistent severe biventricular dysfunction with akinetic and severely dilated right ventricle.

**Figure 1:**
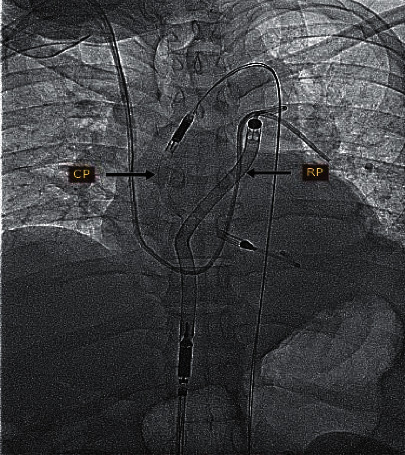
A fluoroscopic image showing the Bi-Pella configuration using the Impella CP and Impella RP axial flow catheters.

**Figure 2:**
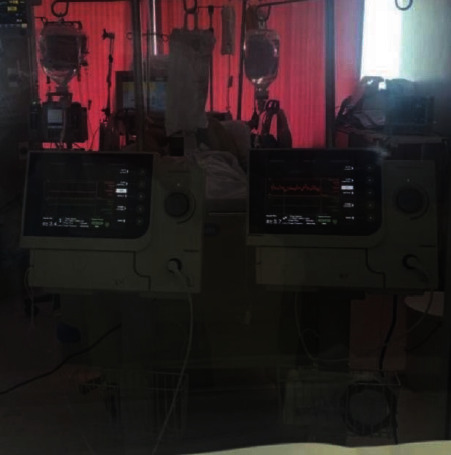
In situ Bi-Pella: consoles for the right and left ventricle.

Based on the clinical presentation and workup findings, the diagnosis of cardiogenic shock secondary to acute inferior myocardial infarction was made. In an attempt to improve his worsening hemodynamic instability, Impella CP (Abiomed, Inc., Danvers, Massachusetts, USA) was deployed and achieved 3.2 L/min of flow at performance level 8 (performance level ranges from P0 to P9), along with successful removal of IABP therapy. The anticoagulation therapy targeted an activated clotting time between 160 and 180 s with heparin. On the next day, his right ventricular function hemodynamic parameters indicated a picture of poor right ventricular function (Table 1), and there was evidence of severe RV dilatation and systolic dysfunction on bedside echocardiogram. Impella RP was then inserted and achieved 3.4 L/min of flow activated at P7 (Figures 1 and 2). Weaning off Impella devices after 24 hours included stepwise reduction of support level to evaluate the recovery of each ventricle. Impella CP was implanted for a total of 5 days and 4 days for the Impella RP. He underwent 7 continuous venovenous hemodialysis (CVVHD) sessions to manage his severe acute kidney injury. A marked improvement of renal and liver function parameters was noticed in the first week after successful explanation of Impella devices. On hospital day 18, the patient was discharged from the coronary care unit, to spend another 14 days in the ward before being discharged home, with LVEF improved to 35%.

## 3. Discussion

**Figure 3:**
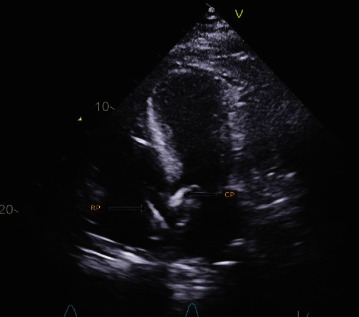
Echocardiographic view of the two pumps in place.

**Figure 4:**
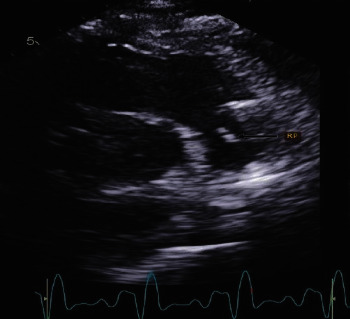
Echocardiographic view of the Impella RP in the right ventricular outflow tract.

CS secondary to biventricular heart failure is associated with significant morbidity and mortality and is considered a real medical challenge [2]. Acute myocardial infarction (AMI) due to cardiogenic shock (AMI-CS) is linked with in-hospital mortality between 33% and 55% [3]. This alarming data have laid the road for the utility of MCS in AMI-CS. Impella CP and RP are examples of microaxial percutaneous MCS devices. They both have different yet similar functions; for the Impella CP, blood is pumped from the left ventricle towards the ascending aorta (Figure 3), and as for the Impella RP, blood is pumped from the vena cava towards the pulmonary arteries (Figure 4) bypassing a failing right ventricle.

With data suggesting that LV dysfunction (LVD) is responsible for the vast majority of shock after AMI [4, 5], results from the IABP-SHOCK II trial, and evidence suggesting improved survival benefits with early application of Impella CP [6], one can understand the reasons behind its increased use in such clinical scenarios. Despite that, it may provide up to 4.0 liters per minute of forwarding flow; some patients, such as in our case, remain hemodynamically unstable. A possible explanation for this is the presence of concomitant severe right ventricular dysfunction (RVD). In fact, in most cases, concurrent RV failure, requiring MCS, is detected after the application of LV hemodynamic support devices or at the initial stages of evaluating a patient presenting with severe biventricular failure.

The pulmonary artery pulsatility index (PAPi), defined as [(systolic pulmonary artery pressure– diastolic pulmonary artery pressure)/central venous pressure], is a reliable predictor of outcome in AMI-CS secondary to RV failure, with values <0.9 suggesting RVD [7]. Poor RV function is strongly associated with a PAPi <0.9 and cardiac power output (CPO) <0.6, especially in patients who are already receiving LV hemodynamic support therapy. Time is a major consideration in RV failure (RVF); ideal management would aim to effectively minimize the delay between the incidence of RVF and implantation of right ventricular hemodynamic support [8]. Due to persistent RV hemodynamic instability, the team elected to insert an Impella RP device, just 24 hours after implantation of Impella CP.

This resulted in significant correction of cardiac index, CVP, SvO2, PAPi, and CPO (Table 1), which strongly correlates with the Recover Right Study [9].

### 3.1. Follow-Up

Shortly after the Impella RP implantation, rapid improvement of hemodynamics was seen in the study patient, as suggested by Table 1. A repeat transthoracic echocardiogram performed 3 months after discharge showed improvement of both LVEF (25%) and RV function.

## 4. Conclusions

We report the first case from the Middle East of AMI complicated by the refractory cardiogenic shock due to biventricular failure that was successfully managed with the early utility of Impella CP and RP (Bi-Pella). The promising survival benefits and the lack of adequate randomized trials to support the use of Impella in AMI-CS warrant urgent additional research on this topic.

## Figures and Tables

**Table 1:** Hemodynamic parameters before and after biventricular impella support.

One day after Impella CP	One day after Impella RP
CVP (mm Hg): 15	CVP: 10
SvO2 (%): 48	SvO2: 55
CI (L/min/m^2^): 1.4	CI: 1.8
CPO: 0.6 (normal > 0.6)	CPO: 0.7
PAPi: 0.73 (normal > 0.9)	PAPi: 1.3
